# G Protein-Coupled Receptors (GPCRs)-Mediated Calcium Signaling in Ovarian Cancer: Focus on GPCRs activated by Neurotransmitters and Inflammation-Associated Molecules

**DOI:** 10.3390/ijms20225568

**Published:** 2019-11-07

**Authors:** Dragoș-Valentin Predescu, Sanda Maria Crețoiu, Dragoș Crețoiu, Luciana Alexandra Pavelescu, Nicolae Suciu, Beatrice Mihaela Radu, Silviu-Cristian Voinea

**Affiliations:** 1 Department of General Surgery, Sf. Maria Clinical Hospital, Carol Davila University of Medicine and Pharmacy, 37-39 Ion Mihalache Blvd., 011172 Bucharest, Romania; 2 Department of Cell and Molecular Biology and Histology, Carol Davila University of Medicine and Pharmacy, 8 Eroii Sanitari Blvd., 050474 Bucharest, Romania; 3 Fetal Medicine Excellence Research Center, Alessandrescu-Rusescu National Institute of Mother and Child Health, Polizu Clinical Hospital, 38-52 Gh. Polizu Street, 020395 Bucharest, Romania; 4 Department of Obstetrics and Gynecology, Alessandrescu-Rusescu National Institute of Mother and Child Health, Polizu Clinical Hospital, 38-52 Gh. Polizu Street, 020395 Bucharest, Romania; 5 Division of Obstetrics and Gynecology and Neonatology, Carol Davila University of Medicine and Pharmacy, Polizu Clinical Hospital, 38-52 Gh. Polizu Street, 020395 Bucharest, Romania; 6 Department of Anatomy, Animal Physiology and Biophysics, Faculty of Biology, University of Bucharest, 91-95 Splaiul Independenţei, 050095 Bucharest, Romania; 7 Life, Environmental and Earth Sciences Division, Research Institute of the University of Bucharest (ICUB), University of Bucharest, 91-95 Splaiul Independenţei, 050095 Bucharest, Romania; 8 Department of Surgical Oncology, Prof. Dr. Alexandru Trestioreanu Oncology Institute, Carol Davila University of Medicine and Pharmacy, 252 Fundeni Rd., 022328 Bucharest, Romania

**Keywords:** ovarian cancer, G-coupled protein receptors, neurotransmitters, inflammation-associated molecules, calcium mobilization, GPCR cross-talk

## Abstract

G-coupled protein receptors (GCPR) involve several signaling pathways, some of them being coupled with intracellular calcium (Ca^2+^) mobilization. GPCRs were involved in migration, invasion and metastasis of different types of cancers, including ovarian cancer. Many studies have discussed the essential contribution of GPCRs activated by steroid hormones in ovarian cancer. However, ovarian cancer is also associated with altered signals coming from the nervous system, the immune system or the inflammatory environment, in which GPCRs are ‘sensing’ these molecular signals. Many studies have been oriented so far on ovarian cell lines (most of them being of human cell lines), and only few studies based on animal models or clinical studies have been devoted to the expression changes or functional role of GPCRs in ovarian cancer. In this paper, we review the alterations of GPCRs activated by neurotransmitters (muscarinic receptors, serotonin receptors, dopamine receptors, adrenoceptors) or inflammation-associated molecules (bradykinin receptors, histamine receptors, chemokine receptors) in ovarian cancer and we discuss their potential as histological biomarkers.

## 1. Introduction

**Figure 1:**
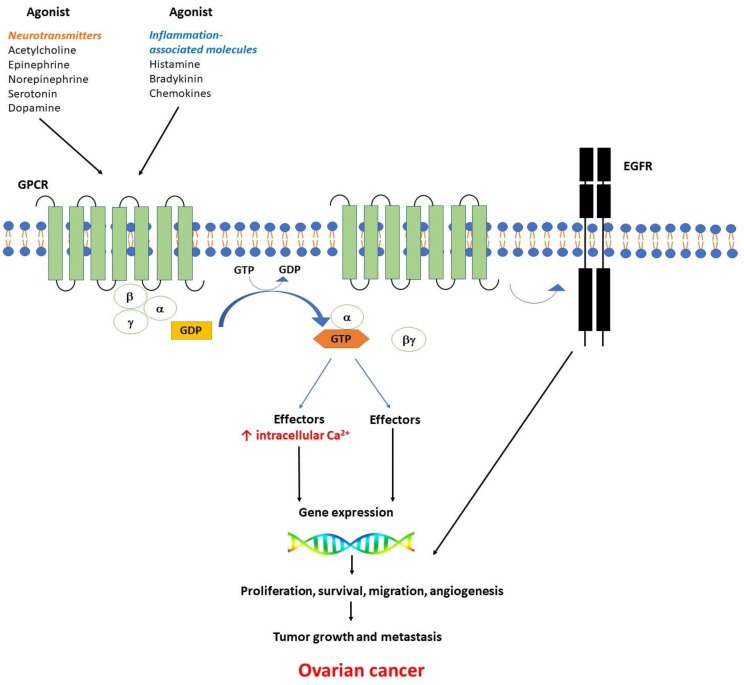
Ligands (agonist) binding to GPCR activate a GTP-binding G protein at the cytoplasmic face of the plasma membrane, followed by the uncoupling of heterotrimeric G proteins into a βγ dimer and the GTP-bound α monomer. Two classes of agonists coupling with GPCRs have been considered: neurotransmitters (acetylcholine, epinephrine, norepinephrine, serotonin, dopamine) and inflammation-related molecules (bradykinin, histamine, chemokines). Multiple downstream effectors are activated by both the βγ dimer and the GTP-bound α monomer, followed by gene transcription and subsequent biological responses. The uncoupled G protein subunits control the activity of many enzymes including kinases, phospholipase C (PLC), and adenylate cyclase to generate second messengers (i.e., intracellular Ca^2+^ increase, ↑ Ca^2+^). There is a cross-talk between GPCR and receptor-tyrosine kinases (RTKs)—see round arrow. Among RTKs, the epidermal growth factor receptor (EGFR) plays a key role in the regulation of important cellular processes. The alteration of the signaling cascades activated by GPCRs may trigger gene expression changes and contribute to cell proliferation, angiogenesis, tumor growth, and metastasis in multiple cancers, including ovarian cancer.

G-protein-coupled receptors (GPCRs) are seven-transmembrane receptors coupled to heterotrimeric G-proteins encoded by the largest gene family in the human genome. At their level an intricate network of transduction cascades are integrated [1]. This large family practically encloses three main categories of receptors: β2 adrenergic–like receptors, glucagon-like receptors, and metabotropic neurotransmitter-like receptors [2]. GPCRs detect a large spectrum of extracellular signals, including ions, peptides, amino acids, or proteins (e.g., neurotransmitters, inflammation-associated molecules, growth factors etc.) [3]. Heterotrimeric guanine-nucleotide-binding regulatory proteins (G-proteins) are composed of three subunits: Gα, Gβ, and Gγ bound in their basal state to guanine diphosphate (GDP). When a ligand binds to the GPCR, GDP is released and replaced by guanosine triphosphate (GTP), while the subunits are dissociated into a βγ dimer and the GTP bound α monomer (Figure 1).

Depending on the α subunit type Gαs, Gαi, Gαq, and Gα11, different downstream effectors are stimulated [4]. Two major classes of agonists coupling with GPCRs will be further considered in this review, neurotransmitters (acetylcholine, epinephrine, norepinephrine, serotonin and dopamine) or inflammation-associated molecules (bradykinin, histamine, and chemokines).

GPCR have been described as key players in multiple cancers, their associated signaling pathways, in some cases GPCR-mediated Ca^2+^ signaling, being involved in the tumorigenesis mechanisms, i.e cell proliferation, migration, invasion and metastasis [5]. Recently, it was shown that there is a cross-talk between GPCR and receptor-tyrosine kinases (RTKs) which integrates intracellular signaling network to control multiple cell functions. Epidermal growth factor receptor (EGFR), a subfamily of RTKs are overexpressed in various tumors and their expression levels correlate with decreased survival rates [6]. It was reported that RTKs are transactivated by GPCRs signaling, either by inducing the cleavage of membrane-bound EGFR-ligand precursors or by directly activating the juxtamembrane tyrosine kinase domain of EGFR (for reviews see [7,8]) (Figure 1). There is a synergistic action of GPCR agonists (i.e., adenosine, angiotensin II, bombesin, bradykinin, cholecystokinin, endothelin, gastrin, lysophosphatidic acid, neurotensin, prostaglandins, vasoactive intestinal peptide, and vasopressin) and agonists of tyrosine kinase receptors, resulting in the induction of cell proliferation [9,10]. Heptahelical receptors are also responsible for mitogenic signaling, including tumorigenesis. The major signal transduction pathways induced by agonist activation of mitogenic GPCRs was broadly reviewed [11].

Multiple studies consider GPCRs as novel pharmacological targets in cancer [12,13]. The contribution of endocrine GPCRs, including reproductive hormone receptors (e.g., G protein-coupled estrogen receptor, follicle-stimulating hormone receptor, luteinizing hormone receptor), hormone receptors involved in gonadotropin release (e.g., kisspeptin receptor, gonadotropin-releasing hormone receptor), or other hormone receptors (endothelin receptors, angiotensin II type 1 receptor) to ovarian cancer progression was extensively reviewed (see [14]). There is a great body of evidence describing the contribution of G-protein coupled estrogen receptors in ovarian cancer, both in cell lines and in patient studies [14,15,16,17,18,19,20,21,22,23], some of them signaling though calcium mediated signaling pathways, while others through mitogen-activated protein kinase signaling pathways. Additionally, androgens and their receptors have been documented to contribute in the etiology and progression of ovarian cancer [24,25], and androgens were demonstrated to influence the expression of G-protein-related genes in ovarian cancer cell lines [26]. Although steroid hormones and GPCRs play a crucial role in ovarian cancer, this topic is beyond the focus of our paper. It is very important to highlight that the mechanisms involved in ovarian cancer staging are more complex than the endocrine alterations. In this context, a systematic review dedicated to the contribution of other GPCRs (i.e activated by neurotransmitters or inflammation-related molecules) in ovarian cancer is largely missing.

Despite the old theory considering that tumors lack innervation [27], recent studies have demonstrated the intense cross-talk between nervous system and cancer cells by multiple neural-related factors [28,29], including neurotransmitters. Indeed, it was demonstrated that neurotransmitters have an essential contribution in tumor angiogenesis, immunity, cancer progress and invasion [30,31,32], and their receptors are upstream regulators in the majority of human cancers [33]. Beside the influence exerted by the nervous system, there is a strong association between inflammation, innate immune system and cancer [34,35,36]. Inflammatory mediators are present both in early stages of cancer, and in cancer cells migration, invasion or metastasis [36], but it is still unclear if tumor inflammatory microenvironment precedes or follows malignancy. Our paper reviews the role of GPCRs activated by neurotransmitters (muscarinic, serotonin, dopamine, and adrenergic receptors) or inflammation-associated molecules (bradykinin, histamine, and chemokine receptors) in ovarian cancer. We are mainly focusing our attention on GPCR-mediated Ca^2+^ signaling, but in some cases, when relevant for the ovarian cancer pathology, we are also discussing the GPCR signaling mediated by other secondary messengers.

## 2. GPCRs Activated by Neurotransmitters in Ovarian Cancer

### 2.1. Muscarinic Receptors in Ovarian Cancer

**Figure 2:**
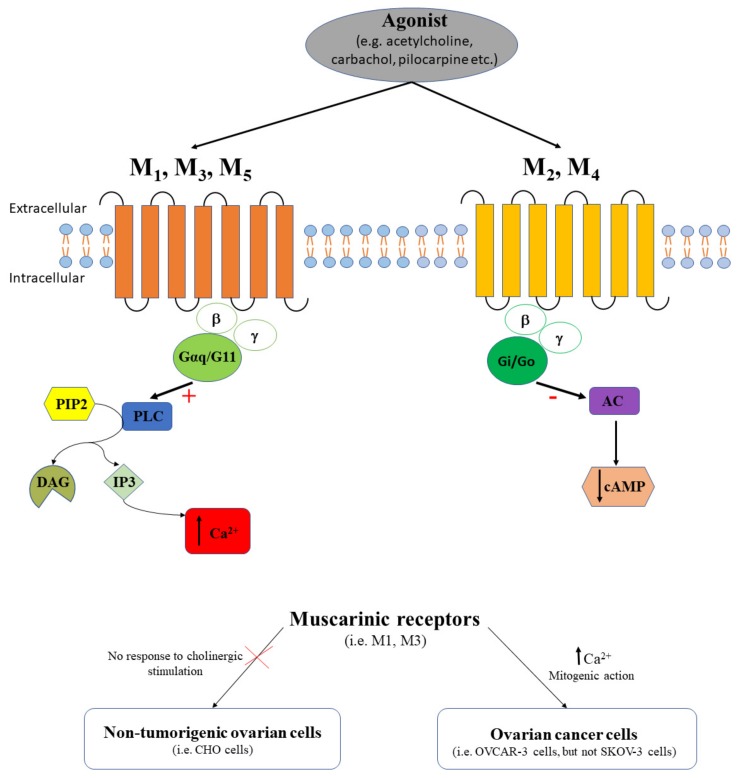
The agonist (e.g., acetylcholine, carbachol, pilocarpine, etc.) binds muscarinic receptors. M1, M3, and M5 receptors couple the PLC signaling pathway with a stimulatory effect (+) that triggers intracellular calcium release (↑ Ca^2+^). M2 and M4 couple the AC signaling pathway with an inhibitory effect (-) that induces cAMP decrease (↓ cAMP). While non-tumorigenic ovarian cells have no response to cholinergic stimulation, in ovarian cancer cells the activation of muscarinic receptors determines the intracellular calcium release (↑ Ca^2+^) and has a mitogenic action.

Muscarinic receptors have five family members, M1, M2, M3, M4 and M5 that belong to the GPCRs. M1, M3 and M5 receptors are coupled with Gq α subunit and activate different downstream effectors in the PLC/IP3/Ca^2+^ pathway. M1 receptor can also couple with Gi (decreases cAMP) and Gs (increases cAMP) α subunit. On the other hand, M2 and M4 receptors are coupled with Gi (decreases cAMP) (Figure 2). 

Acetylcholine is an essential neurotransmitter that regulates multiple functions of the female reproductive system. To date, in physiological conditions, acetylcholine regulates ovarian functions, like ovarian hormone production [37], or growth and differentiation of ovarian follicles [38], and activates muscarinic receptors. In this context, it is important to know which subtypes of muscarinic receptors are expressed in ovarian cells. Studies performed on human ovaries showed that M1 receptors are functionally expressed in the human luteinized granulosa cells, and pirenzepine blocks the intracellular Ca^2+^-mobilization induced by the agonist oxotremorine M [39]. Additionally, M1 receptors were detected in the thecal cells of rat ovarian follicles as well as in cells of the corpus luteum on proestrus day, and it was demonstrated that ovulation demands the activation of these receptors in the left ovary [40]. In vitro studies performed on Chinese hamster ovary (CHO)-K1 cells indicated no response to cholinergic stimulation [41]. Meanwhile, overexpressing M1 receptors or M3 receptors in CHO cells determined a similar intracellular calcium increase in response to pilocarpine, a partial agonist for muscarinic receptors, or to carbachol [41]. The study of Pronin and colleagues [41] is very important to understand that studies on non-tumorigenic ovarian cell lines should be considered with caution, as some of them do not respond to cholinergic stimulation. In myometrium, muscarinic receptors are regulated by steroid hormones (e.g., estrogen receptors, ER); estrogen upregulates M2 (via ERα) and downregulates M3 (ERβ), while progesterone antagonizes estrogen effect on M2 receptor but not against M3 receptor [42]. Similar estrogen effects might be expected to be mediated by muscarinic receptors in ovaries, but further studies are required. To integrate, despite the cholinergic regulation of the ovarian function, only few studies have analyzed in detail the functional role of a specific muscarinic receptor subtype.

Muscarinic receptors play an important role in pathological states of ovaries, i.e., ovarian cancer, and the change in their expression has been analyzed in samples from surgically excised tumors or in tumor cell lines. The presence of muscarinic receptors was demonstrated in multiple ovarian carcinoma cell lines and proved that there is a direct correlation between the Kaplan–Meier analysis of the receptor status in tumors and the survival time in patients with ovarian cancer [43]. However, this study was biased by the use of the non-specific anti-M35 antibody since the authors reported the presence of immunoreactive bands that did not coincide with intense protein bands [43]. Using [3H]quinuclidinyl benzilate (QNB) radioligand assay, the muscarinic receptor expression was also demonstrated in isolated cell membrane fractions from patients with ovarian tumors, cultured tumor cells, and normal ovarian tissue [44]. Interestingly, while muscarinic receptor density is higher in normal human ovarian tissue compared to ovarian tumors, the dissociation constant increases in tumors [44]. On the other hand, muscarinic receptor density in the human ovarian cancer cell line OVCAR-3 is almost triple compared to normal or tumoral ovarian tissue [44]. Due to the differences in muscarinic receptor density between ovarian cancer cell lines vs. tumoral ovarian tissue, it is difficult to compare these data and to give them the appropriate clinical relevance.

The functional status of the muscarinic receptors and the contribution GPCR-mediated Ca^2+^ signaling in ovarian cancer were also described by employing various agonists and antagonists. To date, Batra et al. demonstrated that muscarinic receptors are functionally expressed in human ovarian tumors and in SKOV-3 cells by analyzing the competition QNB vs atropine, or QNB vs carbachol [44]. Moreover, the authors proved that M3 receptors are the predominant receptor subtype expressed in normal ovaries and ovarian tumors by applying specific M3 antagonists, like pirenzepine or AF-DX 116 [44]. Comparing different cell lines, it was found that while human ovarian cancer cell line OVCAR-3 expressed M3 receptors, this receptor subtype was absent in SKOV-3 cell line [44]. Based on the study of Batra and colleagues [44], we should highlight the fact that despite the role attributed to M3 receptors in ovarian cancer, some ovarian cancer cell lines (e.g., SKOV-3) are missing these receptors and studies based on different tumor cell lines should be interpreted with caution. 

Drawing a parallel with some pharmacological studies that demonstrated the regulatory effect of M3 agonists/antagonists on tumor growth in colon and lung cancer, it was hypothesized that M3 antagonists might be also promising candidates for tumor growth inhibition in ovarian cancer [45]. Studies have demonstrated so far only the effects of non-specific muscarinic receptor agonists/antagonists in ovarian cell lines, without testing any specific M3 agonists/antagonists. For example, carbachol, an agonist of muscarinic receptors, increased Ca^45^ uptake by 25% in human ovarian cancer OVCAR-3 cells but had no effect in non-tumorigenic CHO cells [46]. Additionally, carbachol-induced intracellular Ca^2+^ release was partially blocked by the addition of the pertussis toxin or the phorbol ester PMA (phorbol myristate acetate) [46]. Meanwhile, atropine (antagonist of muscarinic receptors) blocked the carbachol-induced cell proliferating effect in OVCAR-3 cells [46]. The authors concluded that mitogenic action of carbachol in ovarian cell lines was mediated through intracellular calcium release that, in turn, promoted DNA synthesis and cell growth [46]. Additional studies showed that verapamil, an L-type Ca^2+^ channel antagonist, and [3,4,5-trimethoxybenzoic acid 8-(diethylamino)octyl ester] (TMB-8), a potent non-competitive antagonist of nicotinic acetylcholine receptors, blocked partially or totally the carbachol-stimulated release of Ca^2+^ from intracellular stores [46]. The authors considered that the effects of verapamil and TMB-8 where rather due to the inhibition of cholinergic receptors than to the suppression of the intracellular calcium release [46]. Unfortunately, additional studies on animal models investigating the role of muscarinic receptors in ovarian cancer are missing, and would be needed to further elucidate the real effect of muscarinic agonists/antagonists on ovarian tumor growth.

To resume, muscarinic receptors are functionally expressed both in human ovarian tumors and in the cancerous cell lines, but not in non-tumorigenic ovarian cells (Figure 2). In most investigated ovarian cancer cell lines, M3 was the predominant functional subtype, and this fact suggests that the GPCR-mediated Ca^2+^ signaling pathway plays a major role in ovarian cancer. This hypothesis is reinforced by the reported increase of carbachol-stimulated Ca^45^ uptake in ovarian cancer cells vs. normal cells [46]. As some muscarinic receptors subtypes are not expressed in specific human ovarian cell lines [46], caution should be taken when extrapolating results from cell lines to animal models and/or human ovarian tissue samples. Existing differences between tumoral and normal ovarian tissue, in terms of muscarinic receptors density and dissociation constant [44], or in Ca^45^ uptake [46], should not contribute to discriminating muscarinic receptors as possible histological markers in our search to distinguish benign from malignant ovarian tumors. It is obviously clear that further studies targeting specific subtypes of muscarinic receptors should be done in near future. 

### 2.2. Adrenergic Receptors in Ovarian Cancer

**Figure 3:**
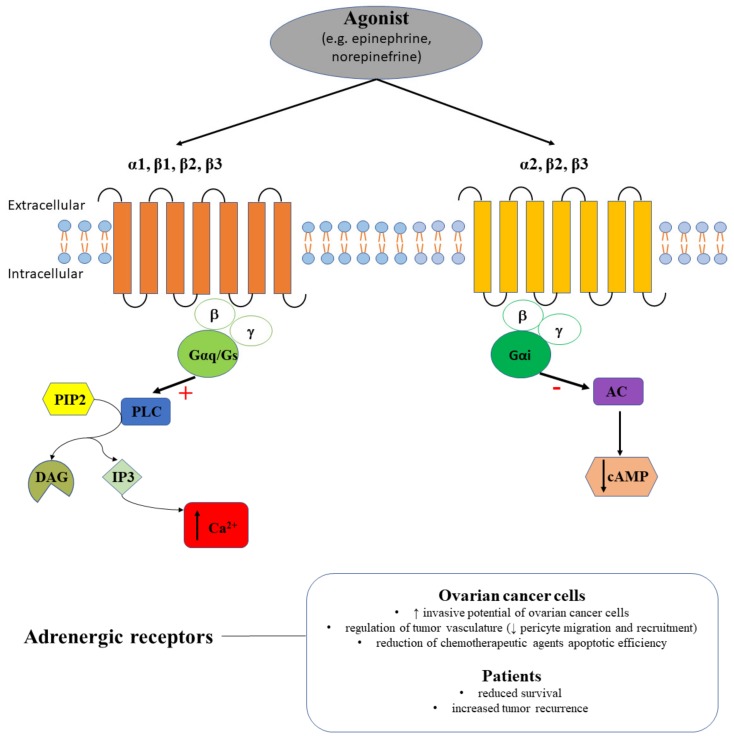
The agonist (e.g., epinephrine, norepinephrine) binds adrenergic receptors. α1, β1, β2, and β3 receptors couple the PLC signaling pathway with a stimulatory effect (+) that triggers intracellular calcium release (↑ Ca^2+^), while α2, β2, and β3 couple the AC signaling pathway with an inhibitory effect (-) that induces cAMP decrease (↓ cAMP). Adrenergic receptors are contributing to the invasive potential of ovarian cancer cells and to increased ovarian tumor recurrence in patients.

Apoptosis, proliferation, and angiogenesis are regulated in physiological conditions by adrenergic receptor signaling pathways. Adrenaline (epinephrine, E) and noradrenaline (norepinephrine, NE) are the endogenous agonists for adrenergic receptors that are coupled to GPCRs. Adrenergic receptors are classified into α-adrenoreceptors (i.e., α1 couples to Gq receptors and its activation determines an increase in intracellular Ca2+; α2 couples to Gi receptors and its activation decreases the cAMP followed by a drop of neurotransmitter release) and β-adrenoreceptors (β1 couples to Gs receptors, β2 and β3 couples to Gs and Gi receptors, and their activation increases cAMP) (Figure 3). 

Catecholamines are essential neurotransmitters that were shown to contribute to the signaling alterations in cancer, in particular in ovarian cancer. Indeed, NE and E are stress hormones and increase the invasive potential of ovarian cancer cells, while regarding the cortisol effect on tumor invasiveness the results were dependent on the tested ovarian cell line [32]. 

In a clinical study based on 237 ovarian cancer cases, 19% of the samples were immunopositive for β2-adrenergic receptors [47]. The same study proved positive correlations between β2-adrenergic receptors-positive tumors and anxiety/depression symptoms, lifetime ovulatory years or oral contraceptive use, but not with ovarian cancer mortality [47]. The contribution of β2-adrenergic receptors to the stress-induced effects on ovarian cancer was reinforced by the Nagaraja and colleagues that demonstrated an increased prostaglandin E2 (PGE2) in patients with depression or in mice subjected to restraint stress following adrenergic stimulation ADRB2-Nf-kB-PTGS2 axis [48].

In a recent study, Modzelewska et al. analyzed the alteration of β-adrenergic receptors in the mechanism of uterine contraction. β-adrenoceptor agonists (e.g., BRL 37344, CL 316243 or ritodrine) had a dose-dependent effect and diminished spontaneous myometrial contractions, in control, endometrial, and cervical cancer groups [49], while their cumulative administration failed to relax the myometrial strips from ovarian and synchronous ovarian–endometrial groups in opposition to the control group [49]. Additionally, administration of β-adrenoceptor antagonists (e.g., propranolol, SR 59230A, or butoxamine) was followed by varied effects on spontaneous uterine contractility when administered cumulatively with β2- or β3-adrenoceptor agonists, depending on the co-administered agonist or on the type of cancer in each examined group [49].

Beside the direct effect on ovarian cancer cells, it was also demonstrated that NE regulates tumor vasculature by acting on the pericytes and endothelial cells. NE decreased mouse 10T1/2 pericyte migration and recruitment, and in combination with dopamine enhanced this effect [50]. In vitro experiments suggested that NE could also influence the chemotherapy efficacy in ovarian cancer. Pre-exposure to NE of HeyA8 or SKOV3ip1 cells prevented chemotherapy-induced apoptosis by paclitaxel and cisplatin [51]. Additionally, exposure of ovarian cancer cells to the β-adrenoceptor agonist isoproterenol determined the same reduced apoptotic efficacy of both chemotherapeutic agents tested, while exposure to β-adrenoceptor antagonist propranolol reverted the effect [51]. In other studies, propranolol blocked the NE-induced invasiveness or interleukin-6 (IL-6) production in the ovarian cancer cell line SKOV-3 [32,52]. 

Only few studies documented the contribution of α-adrenergic receptors in ovarian cancer. To date, α1B adrenergic receptor, but not α2C and β2 receptors, expression was considered to be associated with reduced survival and increased tumor recurrence in patients with endometrioid ovarian cancer [53]. 

Taking into account that β2- or β3-adrenergic receptors have distinct functional alterations in ovarian, endometrial, and cervical cancer [49], pharmacological planning should consider these differences, as some patients are affected by many types of cancer. On the other hand, these functional differences should be further analyzed in terms of expression to assess to opportunity of using β-adrenergic receptors as histological markers. Although prematurely, judging that propranolol increased chemotherapy-induced apoptotic efficiency and blocked invasiveness in ovarian cancer cells, reverting the NE effect [32,51], we can consider in the future the possibility of a combined therapy using chemotherapeutic agents and propranolol to potentiate the antioncogenic effects.

### 2.3. Serotonin Receptors in Ovarian Cancer

**Figure 4:**
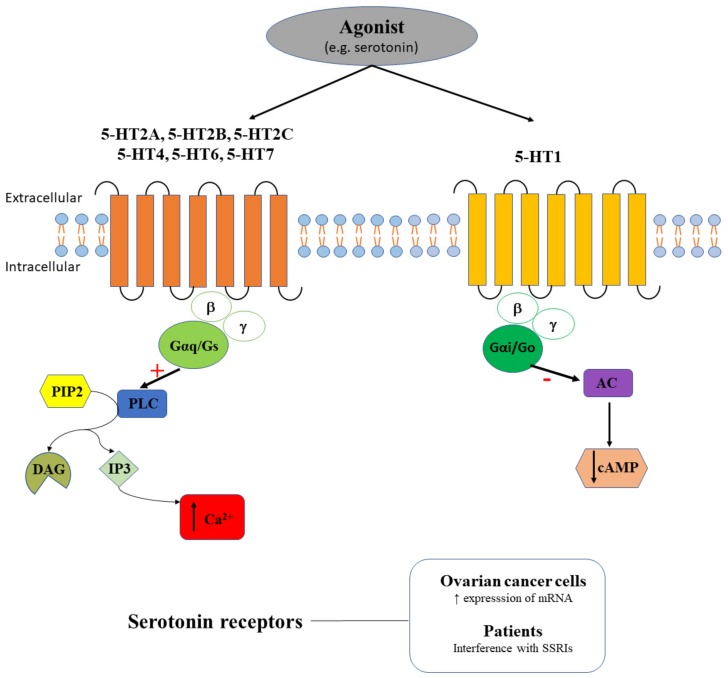
The agonist (e.g., serotonin) binds serotonin receptors. 5-HT2A, 5-HT2B, 5-HT2C, 5-HT4, 5-HT6, and 5-HT7 receptors couple the PLC signaling pathway with a stimulatory effect (+) that triggers intracellular calcium release (↑ Ca^2+^), while 5-HT1 couples the AC signaling pathway with an inhibitory effect (-) that induces cAMP decrease (↓ cAMP). Serotonin receptors have increased mRNA expression in ovarian cancer cells and interfere with SSRI effects in ovarian cancer patients.

Serotonin receptors, except 5-HT3, are coupled to different G proteins (i.e., Gαi/o, Gαs, Gαq/11). In particular, 5-HT2A, 5-HT2B, or 5-HT2C receptors bind to Gαq/11 proteins that activate PLC, increase the production of inositol-trisphosphate (IP3) or diacylglycerol (DAG) and trigger the intracellular Ca^2+^ release [54] (Figure 4). Additionally, some serotonin receptors (i.e., 5-HT4, 5-HT6, 5-HT7) bind to Gαs proteins converting ATP to cAMP, that interacts with protein kinase A (PKA) and regulates intracellular Ca^2+^ [54]. Serotonin has multiple physiological roles, being involved in the regulation of mood and social behavior [55], bone metabolism [56], lactation [57], feeding and satiety [58], sexual desire [59], sleep [60], brain-gut-microbiome axis [61], memory [62] etc.

Alterations of serotonin levels have been associated with multiple pathologies including cancer [63,64]. Patients with malignant tumors have higher serotonin serum levels compared to those with benign tumors [65]. Commonly, oncologic patients are affected by depression and the administration of antidepressants (e.g., selective serotonin reuptake inhibitors - SSRIs, serotonin norepinephrine-reuptake inhibitors and tricyclic antidepressants) is required to regulate serotonin levels. A greater decrease in plasma serotonin levels is generally considered to be associated with an effective SSRI antidepressant treatment [66]. In ovarian cancer patients, SSRI administration was associated with decreased time to disease recurrence, but not with overall survival [67]. Moreover, administration of serotonin or sertraline (SSRI) in an orthotopic mouse model of ovarian cancer determined an increase in tumor weight [68]. 

Therefore, a greater attention should be devoted to the role played by serotonin-associated signaling pathways in all cancers including ovarian cancer, in particular to the involvement of serotonin receptors. A number of studies showed that indeed, serotonin receptors expression undergoes changes in ovarian cancer cells. It was demonstrated that the majority of ovarian cancer cell lines express serotonin receptors [67], but have distinct patterns of expression. Hence, the mRNA 5-HT2A levels are elevated in ovarian cancer cells compared to normal ovarian cells [67], while the expression of 5-HT1A, 5-HT1B, 5-HT2B and 5-HT4 receptors were found elevated in ovaries, in benign and borderline tumors (i.e., epithelial fraction), in non-invasive cancer cells, but strongly decreases in invasive cancer cells [68]. Positive immunoreactivity for 5-HT2B was also detected in the stromal ovarian tissue and in the ovarian blood vessels [68]. 

To resume, the differences observed between benign and malign ovarian tissue in terms of serotonin receptors expression [67,68] might be a good starting point to consider these receptors as histological marker candidates for ovarian cancer in surgical excisional specimens. Moreover, quantifying serotonin levels in serum can be considered as an important biomarker for the prognosis of various types of cancer, including ovarian cancer, even if this biomarker would lack specificity.

### 2.4. Dopamine Receptors in Ovarian Cancer

**Figure 5:**
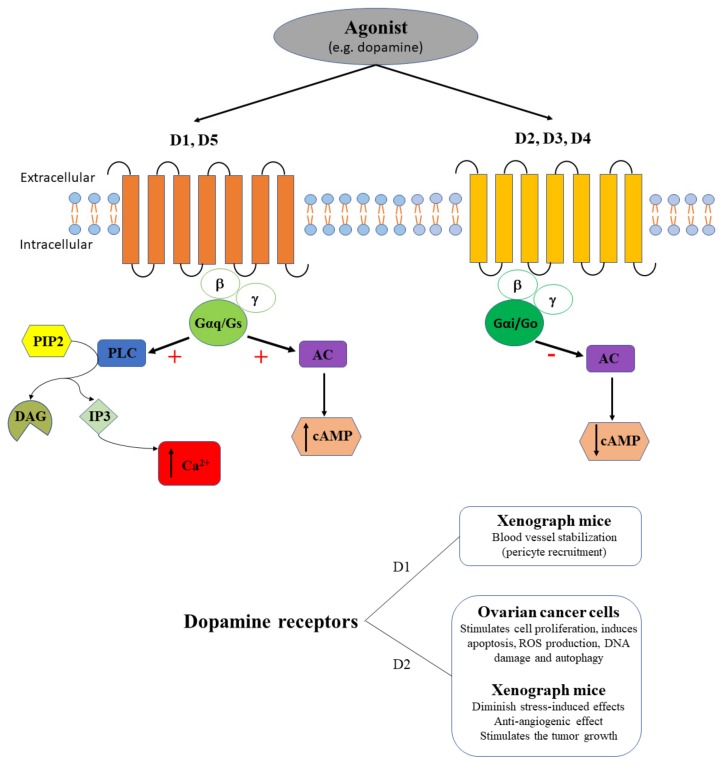
The agonist (e.g., dopamine) binds dopamine receptors. D1 and D5 receptors couple the PLC signaling pathway with a stimulatory effect (+) that triggers intracellular calcium release (↑ Ca^2+^), and couple AC with a stimulatory effect (+) that induces cAMP increase (↑ cAMP), while D2, D3, and D4 couple the AC signaling pathway with an inhibitory effect (-) that induces cAMP decrease (↓ cAMP). D1 receptors contribute to blood vessel stabilization in xenograph mice. D2 stimulate ovarian cancer cells proliferation, apoptosis, ROS production, DNA damage and autophagy and diminish stress-induced effects, has an anti-angiogenic effect, and stimulates tumor growth in xenograph mice.

It is common knowledge that dopamine binds to dopamine receptors (D, part of the GPCR family), exerting stimulatory effects on adenylate cyclase through D1 and D5 that increase cAMP release, while exerting inhibitory effects through D2, D3 and D4, that inhibit the formation of cAMP (Figure 5). Dopamine is an essential catecholamine involved both in physiological (i.e., learning, reward-motivation behavior, emotion, motor control, hormonal regulation, innate immune regulation etc.) and pathological (i.e., Parkinson’s disease, schizophrenia, anxiety, cancer etc.) processes [69,70,71,72,73,74,75,76,77]. Several studies consider D2 as a potential biomarker for different types of cancer [78]. Dopamine and its receptors were demonstrated to be involved in the regulation of tumor cell death, proliferation, invasion, and migration [78]. 

In ovarian cancer, thioridazine (D2 antagonist) has in vivo and in vitro antineoplastic activity [79]. It was demonstrated that thioridazine suppresses cell proliferation, induces apoptosis, ROS production, DNA damage and autophagy (via the AKT/ERK signaling pathway) in SKOV-3 and A2780 cells, and inhibits the growth of SKOV-3 xenografts in nude mice [79] (Figure 5). Interestingly, upregulation of stonin 2 (an adaptor-like protein that regulates endocytic complexes and D2 internalization) is commonly associated with progression and unfavorable prognosis of epithelial ovarian cancer, being correlated with intestinal and intraperitoneal metastasis [80]. Indeed, stonin 2 was upregulated in multiple ovarian cancer cell lines (CAOV3, COV362, COV504, EFO-27, A2780, OVCAR4, SKOV3, and TOV-21G) compared to non-tumorigenic human ovarian surface epithelial cells (HOSEpiC), and in ovarian cancer tissue samples compared to control conditions [80]. In stress conditions, dopamine administration in mice significantly diminished stress-induced effects, including tumor growth and pericyte coverage of tumor vasculature in ovarian cancer [81]. The anti-angiogenic effect was mediated by D2, while the blood vessel stabilization through pericyte recruitment was mediated by D1 [81] (Figure 5). 

In conclusion, despite the role of D2 in ovarian cancer, it is very unlikely to become a specific histological marker for this type of cancer, its expression being altered in many other types of cancer. 

## 3. GPCRs Activated by Inflammation-Related Molecules in Ovarian Cancer

### 3.1. Bradykinin Receptors in Ovarian Cancer

**Figure 6:**
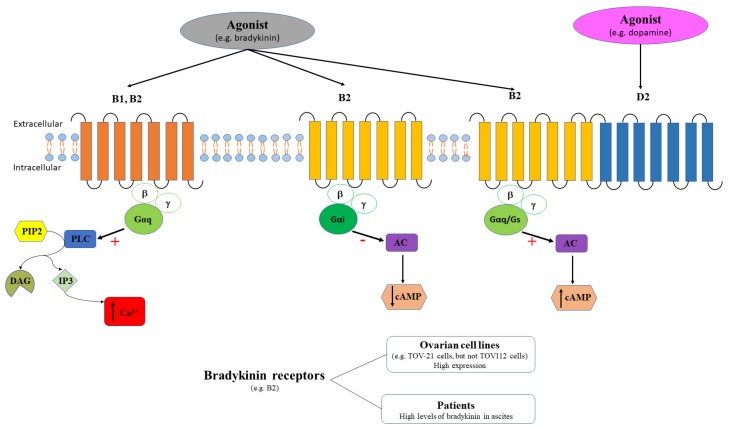
The agonist (e.g., bradykinin) binds bradykinin receptors. B1 and B2 receptors couple the PLC signaling pathway with a stimulatory effect (+) that triggers intracellular calcium release (↑ Ca^2+^), while B2 couples the AC signaling pathway with an inhibitory effect (-) that induces cAMP decrease (↓ cAMP). Additionally, B2 receptors also form dimers with D2 receptors (agonist – dopamine) and couple to AC signaling pathway increasing cAMP [83]. B2 receptors have high expression in some ovarian cell lines, while bradykinin levels are high in ascites of ovarian cancer patients.

Bradykinin receptors (B1 and B2) are members of GPCR family and bradykinin is the main agonist. These receptors are key players in mediating inflammation. While B1 is expressed only in inflammatory conditions, B2 is structurally expressed [82]. B2 receptor is coupled to Gq (stimulates PLC and increases intracellular Ca^2+^) or to Gi (inhibits adenylate cyclase). B2 receptors form dimers with D2 receptors (agonist – dopamine) and couple to AC signaling pathway increasing cAMP [83]. B1 receptor was also described to activate the PLC-signaling pathway that determines intracellular Ca^2+^ mobilization (Figure 6). Although both bradykinin receptors signal through similar pathways, different Ca^2+^ mobilization patterns and dynamics have been described [84,85].

Bradykinin is involved in regulation of multiple physiological processes, including ovulation, oocyte maturation, prostaglandin production [86], vasodilation [87], diuresis [88] etc. Bradykinin is also involved in several pathological states, one of the most prominent being cancer, where bradykinin is considered among the key players that contributes to cell proliferation, migration, invasion, and tumor growth [89]. Several in vitro and in vivo studies demonstrated that bradykinin antagonists may be promising anti-cancer pharmacological agents, in particular in lung and prostate cancers [90]. 

High levels of bradykinin have been detected in ascitic fluids obtained from ovarian cancer patients [91], and the presence of ascites was associated with poor disease prognosis [92]. Yet there are few studies which have analyzed the changes in bradykinin receptors expression in this pathology. To date, the expression and/or function of bradykinin receptors was tested only in ovarian cell lines. For example, the qRT-PCR analysis of the gastrin-releasing peptide receptors expression in several tumor cell lines, including the PEO4 human ovarian cancer cells, indicated a low expression of B2 receptors relative to γ-actin [93]. In two epithelial ovarian cell lines derived from oncologic patients, TOV-21 and TOV-112, the cytotoxic effect of bradykinin receptor antagonist, 2,3,4,5,6-pentafluorocinnamoyl-(o-2,6-dichlorobenzyl)-l-tyrosine-N-(4-amino-2,2,6,6-tetramethyl-piperidyl) amide, BKM-570, was comparable to cisplastin [94]. The same study revealed that B2 was highly expressed in TOV-21 cell line, while no bradykinin receptor expression was detected in TOV-112 cell line [94]. Functional studies employing BK, specific B2 agonist, and des-Arg9-BK, specific B1 agonist, demonstrated that only BK triggered intracellular Ca^2+^ release in TOV-21 cells, while no effect was obtained in TOV-112 cells. The authors concluded that the cytotoxic effect exerted by BKM-570 antagonist could be independent of any bradykinin receptors contribution [94].

To resume, it is still difficult to consider bradykinin receptors as potential histological biomarkers in ovarian cancer surgical resections due to the limited available information. Additionally, in other onco-gynecological pathologies, such as the endometrial cancer, qRT-PCR analysis of patient samples indicated that B1 is upregulated in grade 1 and grade 2 tumors and downregulated in grade 3 tumors, while B2 is upregulated in grade 2 tumors and downregulated in grade 3 tumors [95]. Moreover, among patients experiencing synchronous gynecologic cancers, the most important percentage is represented by synchronous ovarian and endometrial cancers [96,97]. Furthermore, changes in bradykinin expression in the TOV112 ovarian cells [94] might be confusing due to the origin of this cell line derived from patients affected by primary malignant adenocarcinoma and endometrioid carcinoma. Therefore, it is difficult to elaborate a strategy to distinguish between different gynecological cancers based on the histological exam of bradykinin receptors expression. On the other hand, considering that more than one third of ovarian cancer patients have ascites [92], quantifying bradykinin in ascitic fluid can be seen both a marker of inflammation and at the same time gives important information regarding the intraperitoneal dissemination of ovarian cancer.

### 3.2. Histamine Receptors in Ovarian Cancer

**Figure 7:**
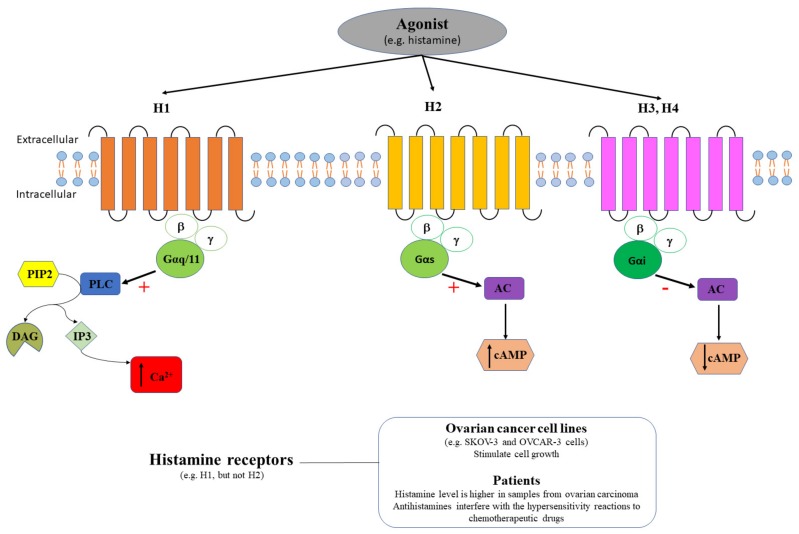
Agonist (e.g., histamine) binds histamine receptors. H1 couple the PLC signaling pathway with a stimulatory effect (+) that triggers intracellular calcium release (↑ Ca^2+^), H2 couple the AC signaling pathway with a stimulatory effect (+) that induces cAMP increase (↑ cAMP), and H3, H4 couple the AC signaling pathway with an inhibitory effect (-) that induces cAMP decrease (↓ cAMP). H1, but not H2, receptors stimulate cell growth in ovarian cancer cell lines (e.g., SKOV-3 and OVCAR-3 cells). Histamine concentration is higher in samples from ovarian carcinoma patients and antihistamines interfere with the hypersensitivity reactions to chemotherapeutic drugs.

Histamine binds to four types of GPCR histamine receptors, H1, H2, H3 and H4. H1 receptors are coupled to Gαq/11 proteins, and subsequently activate the signaling pathway PLC/ IP3/DAG/protein kinase C (PKC) that finally trigger the release of intracellular Ca^2+^. H2 receptors are coupled to Gs proteins and stimulate the cAMP increase. On the other hand, H3 and H4 receptors are coupled to Gi proteins and inhibit cAMP (Figure 7). 

Histamine was described to play an important role in physiological (circadian rhythm, energy homeostasis, cognition, etc.) and pathological (allergic diseases, migraine, neuropsychiatric disorders, etc.) processes [98,99,100,101,102,103]. In cancer, histamine plays a critical role in cell proliferation, migration, and invasion, increasing the resistance to anticancer drug and the expression of markers (i.e., aldehyde dehydrogenase 1) in cancer-initiating cells [104]. Histamine concentration was demonstrated to be higher in samples from ovarian carcinoma (38.47 ± 2.16 µg/g tissue, Figure 7) compared to endometrial carcinoma (19.74 ± 1.21 µg/g tissue) or to normal adjacent tissue (18.54 ± 1.12 µg/g tissue) [105], but no specific analysis of histamine receptors expression was performed. H1 is expressed in multiples types of cancer, including ovarian cancer, and its role in cancer prognosis might be related to the fact that the promoter region of H1 gene contains the binding sites for glucocorticoid receptor (GR), signal transducer and activator of transcription 5A (STAT5A), and c-Myb regulatory transcription factor [106].

The functional expression of histamine receptors and its relationship with ovarian cancer was mainly studied on cell lines. For example, H1-mediated Ca^2+^ mobilization stimulated cell growth in OVCAR-3 cells (Figure 7), but histamine had no effect on non-tumorigenic CHO cells [47]. Meanwhile, pyrilamine (selective H1 antagonist), blocked the histamine-induced cell proliferating effect in OVCAR-3 cells, but had only a minor inhibitory effect on Ca^2+^ influx [47]. In SKOV-3 cells, histamine induced a monophasic rise of intracellular Ca^2+^ both in the presence/absence of external Ca^2+^, and stimulated cell proliferation at high concentrations (micromolar) [107]. Additionally, pyrilamine (H1 antagonist), but not cimetidine (H2 antagonist), was shown to completely abolish the intracellular Ca^2+^ rise induced by histamine [108]. The functional expression of histamine receptors was also demonstrated in endometrioid adenocarcinoma cells, where H1 and H2 are expressed in HEC-1 endometrioid adenocarcinoma cells and histamine upregulates aldehyde dehydrogenase 1, via only H1 but not H2 [104]. 

High levels of histamine exert dual regulatory effects (promoting or inhibiting) tumor growth, including in gynecological tumors; therefore, antihistamines are considered as promising drugs in cancer therapy [108]. However, it is very important to analyze both the effects exerted by antihistamines in ovarian cancer patients and their potential interference with chemotherapeutic agents. The therapeutic use of antihistamines in ovarian cancer should be further confirmed. A population-based case-control study comprising 5556 women in Denmark diagnosed with epithelial ovarian cancer, used antihistamine drugs and found no association with overall ovarian cancer risk [109]. The hypersensitivity reactions to chemotherapeutic agents, involving mast cell and basophils activation, can be monitored in ovarian cancer patients by quantifying the serum levels of tryptase, which is released together with histamine after mast cell activation [110]. The reduction of hypersensitivity reactions to chemotherapeutic drugs (i.e., paclitaxel) in patients with ovarian cancer was also obtained by premedication with pemirolast, an antiallergic agent, that is rather diminishing the release of sensory peptides than inhibiting the histamine release [111]. In a retrospective study, women with ovarian cancer that received premedication with antagonists of H1 and H2, in addition to dexamethasone, had approximately half the risk of carboplatin hypersensitivity reactions compared with patients without premedication and the overall incidence of carboplatin hypersensitivity reactions decreased after addition of premedication [112], Figure 7. In another retrospective study, 14 out of 105 women with ovarian cancer had severe hypersensitivity reactions to paclitaxel administration, regardless premedication with glucocorticoid or H1/H2 antagonists, and chemotherapy was ceased or discontinued [113].

In conclusion, it is difficult to consider histamine receptors as potential biomarkers in the histological exam for the differential diagnosis between ovarian cancer and endometrioid adenocarcinoma. However, current knowledge is limited only to the analysis of few cell lines while data on animal models or patient samples are largely missing. On the other hand, some authors consider histamine receptors as potential pharmacological targets in ovarian cancer. Despite some positive results in the reduction of hypersensitivity reactions to chemotherapeutic agents in ovarian cancer patients [112], antihistamines should be used with caution in these patients as results might be biased by the fact that hypersensitivity reactions to chemotherapeutic agents are commonly associated with tryptase and histamine release from mast cells in the serum [110].

### 3.3. Chemokine Receptors in Ovarian Cancer

**Figure 8:**
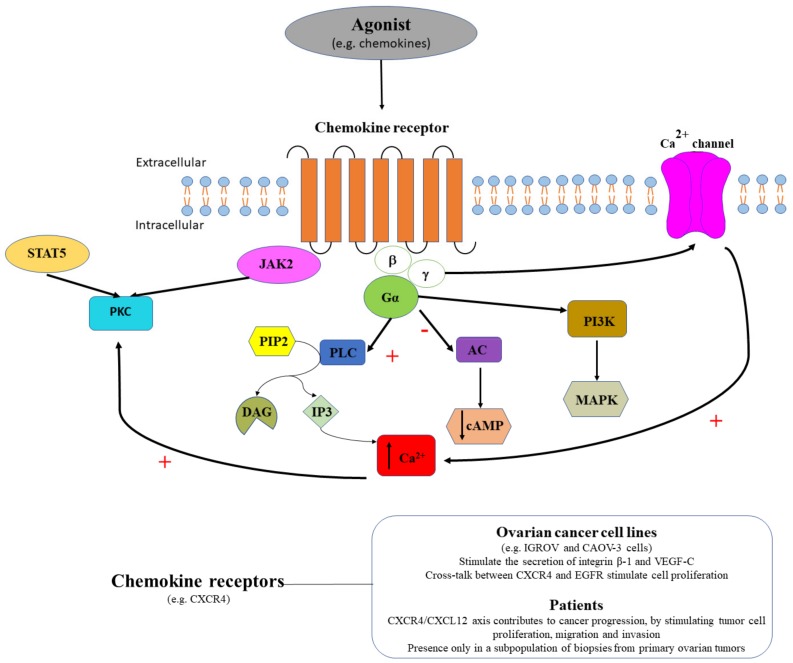
Agonist (e.g., chemokines) binds chemokine receptors, that either couple the PLC signaling pathway with a stimulatory effect (+) that triggers intracellular calcium release (↑ Ca^2+^), or couple the AC signaling pathway with an inhibitory effect (-) that induces cAMP decrease (↓ cAMP), or couple the PI3K signaling pathway stimulating MAPK. CXCR4 receptors stimulate the secretion of integrin β-1 and VEGF-C and their cross-talk with EGFR stimulate cell proliferation in ovarian cancer cell lines. In patients, the CXCR4/CXCL12 axis contributes to cancer progression, by stimulating tumor cell proliferation, migration and invasion. CXCR4 receptors are expressed only in a subpopulation of biopsies from primary ovarian tumors.

Chemokine receptors coupled to G proteins, activate GDP/GTP exchange, followed by dissociation of βγ subunits and activation of phosphoinositide-specific phospholipase Cβ or of phosphoinositide 3-kinase (PI3K), that determines an increase of IP3 or DAG production and the subsequent intracellular Ca^2+^ release or PKC activation (Figure 8). Chemokine receptors are divided into four families: CXC, CC, CX3C and XC chemokine receptors. Chemokines are small signaling molecules that ensure chemotaxis between neighboring cells, being involved in immune surveillance [114]. In pathological conditions, chemokines have been described to be involved in many inflammatory and infectious diseases [115,116,117]. Chemokine receptors have been described as key players in tumor initiation, progression and metastasis in ovarian carcinomas [118]. In this context, chemokine stromal cell-derived factor-1 (CXCL12) and its receptor CXCR4 have been considered as potential therapeutic targets in epithelial ovarian cancer [119], as the in vitro and in vivo blocking (i.e., AMD3100, selective CXCR4 antagonist) of the CXCR4/CXCL12 axis inhibited ovarian cancer progression, by reducing tumor cell proliferation, migration and invasion [119,120]. 

Chemokine receptors expression was demonstrated in ovarian cancer cell lines. In depth, the functional expression of CXCR4 in IGROV and CAOV-3 ovarian cancer cells were demonstrated by the presence of CXCL12-induced Ca^2+^ transients [121]. In six ovarian cancer cell lines (SKOV-3, IGROV, OVCAR-3, CAOV-3, PEO1, and PEO14) the expression of CXCR4 mRNA was demonstrated, but no expression was detected for the other analyzed chemokine receptors (i.e., CCR1, CCR2a, CCR2b, CCR3, CCR4, CCR5, CCR7, CCR8, CXCR1, CXCR2, CXCR3, CXCR5, or CX3CR1) [121] (Figure 8). In CAOV-3 cells, CXCL12 and its receptor CXCR4 stimulated the secretion of integrin β-1 and vascular endothelial growth factor-C (VEGF-C) [122], indicating the involvement of this receptor in the stimulation of tumor vasculature in ovarian cancer. The cross-talk between CXCL12-activated CXCR4 and EGFR was suggested to play an important role in cell proliferation signaling in ovarian carcinoma cell lines [123,124]. Furthermore, CXCR-1 and CXCR-2 were also demonstrated to be strongly expressed in ovarian cancer cell lines, and to activate mitogen-activated protein (MAP) kinase via EGFR, contributing to cell migration and proliferation [125]. In addition, a calcium-dependent phosphorylation mechanism in the IL-8 induced EGFR transactivation was demonstrated, by exposing SKOV-3 cells to A23187 (Ca^2+^ ionophore); BAPTA (intracellular Ca^2+^ chelator) reverted phosphorylation, while EGTA (extracellular Ca^2+^ chelator) had no effect [125].

Similarly, chemokines receptors expression was demonstrated in surgical specimens or biopsies from primary ovarian tumors. To date, CXCR4 mRNA was detected in a cell subpopulation (1 to 20%) of biopsies from primary ovarian tumors [120]. An extensive histological analysis of primary ovarian tumors upon laparotomy, indicated CXCL12 as an independent predictor of poor survival [126]. Moreover, high levels of CXCL12 were detected in ascitic fluid from patients with ovarian cancer [121], and some authors proposed the CXCR4/CXCL12 pair as a molecular target in ovarian cancer [127]. 

To conclude, it is premature to consider chemokine receptors as reliable histological markers or pharmaceutical targets. First of all, not all biopsies from primary ovarian tumors were positive for these receptors [121,126], and the histological diagnosis would be relevant only for some patients. Secondly, chemokines receptors cross-talk with EGFR [123,124] creates difficulties in a successful drug targeting of these receptors. However, corroborating the high levels of CXCL12 in ascitic fluid that indicate poor survival [121] with poor disease prognosis due to ascites presence [92], we can assume that quantifying CXCL12 levels in ascitic fluid might represent a good biomarker in ovarian cancer. 

## 4. Conclusions

GPCRs are considered as important pharmacological targets in multiple cancers, including ovarian cancer. Current review was focused mainly on GPCR-mediated Ca2+ signaling, a subject that until now received very little attention, but which should be of relevance for the ovarian cancer pathology. The most important contributions in ovarian cancer proliferation, migration, invasion and metastasis is given by GPCRs activated by neurotransmitters and by inflammation-associated molecules. We resumed here the results of most relevant studies in Table 1.

Unfortunately, the data available in the literature on this subject are hampered by the fact that most of them are at the stage of experimental studies. Most such studies were performed on human ovarian cell lines, and only few studies are based on animal models, and fewer are related with clinical studies. This represent an inconvenient in determining the relevance of these GPCRs as possible biomarkers or as potential future targets for molecular therapy. Considering the importance of the control exerted by neurotransmitters in tumorigenesis and angiogenesis, future studies should aim to create more appropriate preclinical models. These models are needed to create the premises to draw reliable conclusions which will allow us to validate which GPCR is aberrantly expressed in ovarian cancer and how the downstream products resulting from the signaling cascade can be influenced. Additionally, when considering new intraoperative histological markers (such as GPCRs), the revised guidelines of International Federation of Gynecology and Obstetrics (FIGO)’s staging classification for cancer of the ovary, fallopian tube, and peritoneum should be followed [128].

Therefore, in our search for new potential biomarkers, the discovery of data about GPCR-mediated pathways will allow probably in the not too distant future a more carefully patient selection. Moreover, it will open new avenues in defining more effective combined treatments and yet untrickable association between chemotherapeutic drugs and GPCRs agonists/antagonists. 

Some general conclusions may be also summarized: (1) GPCRs are functionally expressed in some ovarian cancer cells, (2) GPCRs have an expression increase in ovarian tumor cells and/or tumor vasculature; (3) GPCRs contribute to cell proliferation, migration and invasion and in tumor differentiation; and (4) specific GPCRs agonists stimulate ovarian cancer development, while GPCRs antagonists reverse the effects. Some ovarian cancer lines are insensitive to agonist (e.g., neurotransmitter or inflammation-associated molecules) stimulation, indicating the absence of functional expression of these GPCRs. Therefore, translational studies ovarian cancer cell lines → mouse xenograph models → patients with ovarian cancer should be done carefully, despite the difficulties to choose the most appropriate model for ovarian cancer that also mimics GPCRs alterations.

The contribution of GPCRs in ovarian cancer is complex due to the cross-talk between GPCR receptors and themselves or with other receptors. In general, the cross-talk involving PLC-coupling determined an increase in intracellular Ca^2+^ mobilization [129], while the cross-talk involving PKC-coupling determined a decrease in intracellular Ca^2+^ release [130]. Considering the complexity of GPCRs activation mechanisms, involving signaling cascades and/or cross-talk with different receptors, it is difficult to consider a particular GPCR receptor as a molecular pharmacological target. However, GPCRs alterations represent important biomarkers in ovarian cancer and may represent useful tools in the histopathological exam for precise delimitation of tumorigenesis-affected tissue from normal one. 

## Figures and Tables

**Table 1:** GPCRs activated by neurotransmitters and by inflammation-associated molecules in ovarian cancer.

GPCR	Receptor	Ca2+ Signaling ^#^	Type of Sample	Expression/Functional Status of GPCRs in Ovarian Cancer Analyzed in Cell Lines or Patient Samples
**GPCRs Activated by Neurotransmitters**				
Muscarinic receptors	N/A	Yes	OVCAR-3 cells	Carbachol increases Ca^45^ uptake by 25% of the ovarian cancer cells [46]
	N/A	Yes	OVCAR-3 cells	Atropine blocks the carbachol-induced ovarian cancer cell proliferating effect [46]
	N/AM3	Yes	Normal human ovary Human ovarian tumors SKOV-3 cells OVCAR-3 cells	Muscarinic receptors are functionally expressed in ovarian cancer cells, M3 being predominant [44]
Adrenergic receptors	β2	No	Human ovarian tumors	19% of the samples were immunopositive for β2-adrenergic receptors [47]
		No	Skov3-ip1 cells HeyA8 cells	NE, isoproterenol, and terbutaline stimulate PGE2 production, contributing to cancer cell migration and invasion [48] Silencing prostaglandin-endoperoxide synthase 2 reduces the NE-induced cancer cell migration and invasion [48]
	β2β3	No	Myometrial strips from ovarian cancer patients or patients with ovarian cancer in combination with endometrial cancer	β-adrenoceptor agonists diminished spontaneous uterine contractility, but had contradictory effects in cumulative administration [49] β-adrenoceptor antagonists caused varied effects on spontaneous uterine contractility when co-administered with β-adrenoceptor agonists [49]
	Β	No	HeyA8 and SKOV3ip1 cells	Pre-exposure to NE prevents chemotherapy-induced apoptosis [51] Isoproterenol reduces apoptotic efficacy of chemotherapeutic agents, while propranolol reverts the effect [51]
	Β	No	HeyA8 and SKOV3ip1 cells	Propranolol reverts the NE-induced IL-6 production [52]
	α1B	Yes	Endometrioid ovarian tumors	α1B expression is a marker of reduced survival and increased tumor recurrence [53]
Serotonin receptors	5-HT1A 5-HT1B 5-HT1D 5-HT1E 5-HT2A 5-HT2B 5-HT4	No No No No Yes Yes Yes/No	A2780-CP20, SKOV3, HEYA8, 2774, ES2, TOV112D, OV90, SW626, UWB1.298 and CaOV3 cells	5-HT1A, 5-HT1B, and 5-HT1D have a low expression [67] 5-HT2A has a high expression; DOI increases clonogenic survival [67] 5-HT1E is expressed only in 2774 and CaOV3 cells [68] 5-HT1A, 5-HT1B, 5-HT2B and 5-HT4 expression strongly decreases in invasive cancer [68]
Dopamine receptors	D1	No	SKOV3ip1 and HeyA8 tumor-bearing nude mice	Butaclamol has no efficacy against the inhibitory effect of dopamine on stress-mediated tumor growth [50] SKF 82958 increases the extent of pericyte coverage in the tumoral tissue [50]
	D2	No	SKOV3 and A2780 cells	Thioridazine suppresses cell proliferation, induces apoptosis, ROS production, DNA damage and autophagy [79]
		No	SKOV3 xenografts in nude mice	Thioridazine inhibits tumor growth [79]
		No	CAOV3, COV362, COV504, EFO-27, A2780, OVCAR4, SKOV3, and TOV-21G cells	Upregulation of stonin 2 [80]
		No	Epithelial ovarian cancer patient samples	Upregulation of stonin-2 is associated with progression and unfavorable cancer prognosis, being correlated with intestinal and intraperitoneal metastasis [80]
		No	SKOV3ip1 or HeyA8 tumor-bearing nude mice	Eticlopride suppresses the inhibitory effect of dopamine on tumor growth and angiogenesis in stress conditions [81] Parlodel has no effect on the pericyte coverage in the tumoral tissue [81]
**GPCRs Activated by Inflammation-Associated Molecules**				
Bradykinin receptors	B2	Yes/No	PEO4 cells	Low expression [93]
		Yes	TOV-21 cells TOV-112 cells	Prominent expression in TOV-21 cells [94] BKM-570 effect is comparable to cisplastin [94] BK, but not des-Arg9-BK, triggers intracellular Ca^2+^ release in TOV-21 cells [94]
Histamine receptors	H1	Yes	OVCAR-3 cells	H1-mediated Ca^2+^ mobilization stimulates cell growth [47] Pyrilamine blocks the histamine-induced cell proliferating effect [47]
		Yes	SKOV-3 cells	Pyrilamine, but not cimetidine, completely abolish the intracellular Ca^2+^ rise induced by histamine [106]
	N/A	Yes	SKOV-3 cells	Histamine induces a monophasic rise of intracellular Ca^2+^ both in the presence/absence of external Ca^2+^ [107] Histamine stimulates cell proliferation at high concentrations (micromolar) [107]
Chemokine receptors	CXCR1 CXCR2	Yes/No	SKOV-3 cells	Activate MAP kinase via EGF receptor; stimulate cell migration and proliferation [125]
	CXCR4	Yes	CAOV-3 cells	Stimulate secretion of integrin beta-1 and VEGF-C [122]
		Yes	IGROV cells CAOV-3 cells Human ovarian tumors	Strong CXCR4 receptors expression in cell lines and human ovarian tumors [121] Blocking CXCR4 receptors with AMD3100 inhibits ovarian cancer progression [119,120]

^#^ Information in this column indicates the commonly signaling pathway activated by each receptor and is not correlated with the methodology used in the indicated references.
